# AAV retinal transduction in a large animal model species: Comparison of a self-complementary AAV2/5 with a single-stranded AAV2/5 vector

**Published:** 2009-09-11

**Authors:** S.M. Petersen-Jones, J.T. Bartoe, A.J. Fischer, M. Scott, S.L. Boye, V. Chiodo, W.W. Hauswirth

**Affiliations:** 1 Department of Small Animal Clinical Sciences, Michigan State University, East Lansing, MI; 2 Department of Neuroscience, The Ohio State University, Columbus, OH; 3 Department of Ophthalmology, University of Florida, Gainesville, FL

## Abstract

**Purpose:**

To compare self-complementary (sc) and single-stranded (ss) adeno-associated viral 2/5 (AAV2/5) vectors for retinal cell transduction in the dog when delivered by subretinal injection.

**Methods:**

ScAAV2/5 and ssAAV2/5 vectors encoding enhanced green fluorescent protein (GFP) under control of the chicken beta actin promoter were prepared to the same titer. Equal amounts of viral particles were delivered into the subretinal spaces of both eyes of two dogs. In each dog, one eye received the scAAV2/5 and the other the ssAAV2/5. In vivo expression of GFP was monitored ophthalmoscopically. The dogs were sacrificed, and their retinas were examined by fluorescent microscopy and immunohistochemistry to determine GFP expression patterns and to assay for glial reactivity.

**Results:**

GFP expression in the scAAV2/5 injected eyes was detectable at a much earlier time point than in the ssAAV2/5 injected eyes. Expression of GFP was also at higher levels in the scAAV2/5-injected eyes. Expression levels remained stable for the seven month duration of the study. The types of cells transduced by both vectors were similar; there was strong reporter gene expression in the RPE and photoreceptors, although not all cones in the transduced area expressed GFP. Some horizontal and Müller cells were also transduced.

**Conclusions:**

When delivered by subretinal injection in the dog, scAAV2/5 induces faster and stronger transgene expression than ssAAV2/5. The spectrum of retinal neurons transduced is similar between the two vectors. These results confirm in a large animal model those previously reported in the mouse. ScAAV2/5 shows promise for use in the treatment of conditions where a rapid transgene expression is desirable. Furthermore, it may be possible to use a lower number of viral particles to achieve the same effect compared with ssAAV2/5 vectors.

## Introduction

The many different forms of retinal dystrophy that spontaneously occur in the dog have made this species an extremely valuable large animal model for therapeutic interventions. One example is the RPE65 mutant dog model of Leber Congenital Amaurosis that has been used to evaluate gene therapy approaches before human clinical trials [1,2]. In addition to the RPE65 mutant dog there are dog models of autosomal recessive retinitis pigmentosa due to different gene mutations [3-5], autosomal dominant retinitis pigmentosa [6], X-linked RP [7], cone-rod dystrophies [8,9], and achromatopsia [10]. Several of these models are being used to develop and test gene therapy approaches that are ultimately aimed for use in human patients. Transduction of photoreceptor or retinal pigment epithelial cells has been successfully achieved with adeno-associated viral (AAV) vectors, partly due to their ability to transduce nondividing cells, the resulting long-term expression of the transgene, and the lack of problems associated with immune responses [11]. There are some recognized drawbacks to the current AAV vectors. One of which is the presence of a lag-phase before transgene expression following cell infection with the vector. One factor that contributes to the delay in AAV-introduced transgene expression is the requirement for the single-stranded genome to be converted to double-stranded DNA in the host nucleus [12-14]. A lag in transgene expression is problematic when attempting to treat rapidly progressive conditions, where an early onset of transgene expression is particularly important for success of the therapy. One such large animal example is the PDE6A mutant dog model of autosomal recessive RP in which peak levels of rod apoptosis have been detected at about four weeks of age [15]. This rapid loss of photoreceptors has made gene replacement therapy in this model challenging (SPJ, unpublished results). It is likely that similar challenges could be faced when attempting to rescue rod photoreceptors in human RP patients with mutations that result in a rapid loss of rod photoreceptors. Recently self-complementary AAV (scAAV) vectors have been developed that may overcome this rate-limiting step [16]. ScAAV vectors consist of a single-stranded inverted-repeat genome separated by a mutated terminal resolution site. The insert is designed so that the DNA can fold on itself to produce a double stranded DNA version of the transgene, without requiring DNA synthesis. ScAAV vectors have improved transduction efficiency in several tissues, including liver, muscle, CNS, bone marrow and the eye, compared to single-stranded AAV (ssAAV) preparations (see [17] for a review). In mice, a comparison of reporter gene expression in the outer retina following subretinal injection has shown that scAAV2 transgene expression is faster and stronger compared with that achieved by the equivalent ssAAV2 vector [18]. A study comparing multiple AAV serotypes delivered by subretinal injection in mice showed that scAAV2/8 expressing green fluorescent protein (GFP) had faster and stronger expression than the equivalent ssAAV2/8 construct. A comparison between scAAV2/5 and ssAAV2/5 showed that the self-complementary construct induced stronger reporter gene expression, but with a similar time of onset, compared with the equivalent single-stranded construct [19].

This study was undertaken to investigate how efficiently a scAAV2/5 preparation transduces the canine outer retina in comparison to a ssAAV2/5 preparation. It showed that scAAV2/5 results in a faster turn on and stronger transgene expression in the canine retina.

## Methods

### Production of ssAAV2/5 and scAAV2/5

AAV vectors were manufactured and purified by previously described methods [20]. Vector titer was determined by real time PCR, and final aliquots were resuspended in balanced salt solution (Alcon Laboratories, Fort Worth, TX) containing 0.014% Tween-20. The ssAAV and scAAV were type 2/5 vectors carrying the enhanced GFP (eGFP) gene driven by the chicken beta actin promoter. The scAAV2/5 construct had a titer of 0.5×10^12^ viral particles per milliliter (vp/ml) and the ssAAV2/5 construct was prepared to the same titer. Our previous unpublished studies showed that this titer of sAAV2/5 delivered by subretinal injection in the dog results in outer retinal GFP expression, which can be detected in vivo by our imaging system.

### Subretinal injections

Two normal adult crossbred dogs (a 35-month-old male and a 29-month-old female) that were produced as part of a breeding program at Michigan State University were used in the experiment in compliance with the ARVO statement for the Use of Animals in Ophthalmic and Vision Research and approved by the institutional animal care and use committee. Dogs were housed in the College of Veterinary Medicine vivarium, at Michigan State University. They were given ad libitum water and fed a mixture of Hill’s Maintenance (Hill’s Pet Nutrition inc. Topeka, KS) and Purina Pro Plan (Purina-Nestle, St Louis, MO). They were housed under 12 h light:dark cycles. Following pupillary dilation by application of a drop of topical tropicamide 1% ophthalmic drops (Mydriacyl. Alcon Pharmaceuticals, Ft. Wayne, TX), both dogs underwent a full ophthalmoscopic examination (including slit lamp biomicroscopy, indirect ophthalmoscopy and fundus photography) before the study. For subretinal injection the anesthesia protocol used was to premedicate with intramuscular acepromazine maleate (Boehringer Ingelheim Vetmedica Inc., St. Joseph, MO; 0.1–0.3 mg/kg), induce with intravenous thiopental sodium (Pentothal, Hospira Inc., Lake Forest, IL; 6–12 mg/kg given to effect) and maintain with inhaled isoflurane (Isoflo, Abbott Laboratories, North Chicago, IL; 1 to 2%) delivered in oxygen via an endotracheal tube. The eyes were prepared for aseptic surgery by swabbing the ocular surface and surrounding adnexa with a 1:50 dilution of povidone-iodine solution. A sterile fenestrated drape was applied and an eyelid speculum inserted. The eye was manipulated into a primary gaze position by the use of 4–0 silk (Ethicon, Inc., Piscataway, NJ) stay sutures placed in the perilimbal conjunctiva. Under direct observation through an ophthalmic operating microscope (Opmi6. Carl Zeiss Meditech Inc., Dublin, CA) and a Machemer vitrectomy lens (Ocular Instruments, Bellevue, WA) a subretinal injector (RetinaJect. SurModics Inc., Irvine, CA) was used to deliver a subretinal injection [21]. Briefly, the injector was introduced through the pars plana and, under direct observation, was advanced across the vitreal cavity toward the retinal surface. The device had a 39 gauge extendable cannula. This was extended to press on the retinal surface. As the injection was made, the fluid pressure created a retinotomy. The injected fluid passed through the retinotomy into the subretinal space and induced retinal detachment.

In each dog, the left eye was injected with 250 µl of scAAV2/5-GFP vector at 0.5×10^12^ vp/ml. The right eye was injected with 250 µl of ssAAV2/5-GFP at the same concentration of viral particles. Injections were made in the tapetal region in all eyes. Wide-angle fundus images were captured before and after subretinal injection using a digital fundus camera (RetCam II. Clarity Medical Systems, Pleasanton, CA).

Post-operative analgesia was provided by intramuscular administration of 0.01 mg/kg buprenorphine (Torbugesic, Fort Dodge Animal Health, Fort Dodge, IO). Topical broad spectrum and steroid ophthalmic ointment (Neomycyn and Polymyxin B Sulfates and Dexamethasone, Alcon Laboratories, Fort Wayne, TX) was placed in each eye after surgery and three times daily for 4 days.

### Ophthalmoscopic monitoring

The dogs had full ophthalmology examinations the day following surgery and then every two days for the first week after injection and then subsequently two times per week until euthanasia. Fundus images were captured at every examination, and the fluorescein angiography settings of the fundus camera were used to screen for GFP expression. To detect low levels of fluorescence, we enhanced images for contrast and brightness using Photoshop (Photoshop CS2, Adobe Systems Inc., San Jose, CA).

### Immunohistochemistry

Dogs were humanely euthanized with an overdose of intravenous pentobarbital sodium (86 mg/kg, Fatal Plus, Vortech Pharmaceuticals, Dearborn, MI) one at six and the other at seven months following subretinal injections. The globes were immediately removed and slits were made through pars plana into the vitreous. The globes were immersed for 15 min at 4 °C in 4% paraformaldehyde plus 3% sucrose in 0.1M phosphate buffered saline (PBS; 50 mM sodium phosphate, 195 mM NaCl; pH 7.4). The anterior segment was then removed and the posterior eyecup returned to the same fixative for 20 min, then washed three times for 30 s in fresh PBS at 4 °C. The eyecup was placed in PBS plus 30% sucrose for 24 h then immersed in embedding medium (Tissue-Tek OCT compound, Sakura Finetek USA Inc., Torrance, CA).

From the OCT blocks 14 μm sections were cut in a vertical plane through the optic nerve head and thaw-mounted onto Super-Frost^TM^ slides (Fisher Scientific Ltd, Leicestershire, UK), air-dried and stored at −20 °C until use.

Primary antibodies used are listed in Table 1. Sections were incubated for 24 h at 20 °C in a humidified chamber. The slides were washed in PBS, covered with secondary antibody solution, and incubated for 1 h at 20 °C in a humidified chamber. Secondary antibodies included goat-anti-rabbit-Alexa488 and goat-anti-mouse Alexa568/647 (Molecular Probes Inc., Eugene, OR).

**Table 1:** Details of primary antibodies used for IHC

**Antibody**	**Host**	**Target cell**	**Working dilution**	**Source**
Anti-GFP	Rabbit	Cells expressing GFP protein	1:5,000	Dr. Luc Berthiaume, University of Alberta, Alberta, Canada
Human Cone Arrestin	Rabbit	Cone photoreceptors	1:100,000	Dr. Cheryl Craft & Xuemei Zhu, Mary Allen Lab, Doheny Eye Institute, University of Southern California, Los Angeles, CA
Protein Kinase C alpha	Mouse	Rod bipolar cells	1:200	BD Biosciences, PharMingen, Rockville, MD
PSD95	Mouse	Rod spherules and cone pedicles	1:100	Neuromab, University of California Davis, Davis, CA
Calbindin	Mouse	Horizontal cells	1:1,000	Swant Immunochemicals, Bellinzona, Switzerland
Calretinin	Rabbit	Horizontal cells	1:1,000	Swant Immunochemicals, Bellinzona, Switzerland.
GFAP	Rabbit	Astrocytes and reactive Müller glia	1:2,000	DakoCytomation, Carpinteria, CA

Photomicrographs were taken using a Leica DM5000B microscope equipped with epifluorescence and a 12 megapixel Leica DC500 digital camera. Images were optimized for color, brightness, and contrast, and double-labeled images overlaid by using Photoshop^TM^ 6.0. (Adobe Systems Inc.). Confocal imaging was done using a Zeiss LSM 510 system at the Hunt-Curtis Microscopy Facility at The Ohio State University.

## Results

### Rate and strength of GFP expression

Following subretinal injection the retina was found to have reattached by two days postinjection in all eyes. As expected, there was a mild surgically induced intraocular inflammatory reaction in all four eyes that resolved within the first week. Both eyes of one dog showed ophthalmoscopic evidence of mild retinal inflammation with engorgement of retinal vasculature. This resolved within one week. No medical therapy was required.

**Figure 1:**
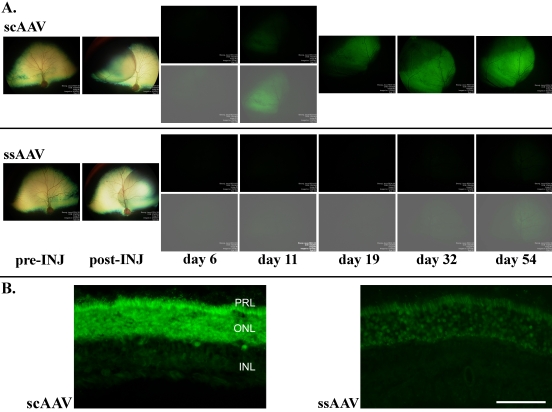
Comparison of GFP expression in scAAV2/5 and ssAAV2/5 injected eyes (dog 1). **A**: Pre-INJ shows the fundus appearance before subretinal injection. post-INJ images were taken immediately after subretinal injection and show the resulting retinal detachment. The images day 6 to day 54 were taken using identical fluorescein angiography settings. The lower images (day 6 and 11 for scAAV2/5 and day 6 to 54 for ssAAV2/5) were adjusted for brightness and contrast to the same degree. GFP expression in the scAAV2/5 injected eye was detectable from day 6 whereas in the ssAAV2/5 injected eye it was not apparent until day 32. GFP expression was stronger in the scAAV2/5 injected eye. **B**: Comparison of GFP fluorescence in retinal cross sections from the center of the injected regions of both eyes of dog 1 (obtained with identical microscope and camera settings). Scale bar equals 50 µm.

GFP expression as monitored ophthalmoscopically had a similar pattern in both dogs. Fluorescence in the scAAV2/5-GFP-injected eyes was apparent much sooner after injection and was notably stronger compared to that seen in the ssAAV2/5-injected eyes. In the scAAV2/5-GFP-injected eyes, faint GFP expression was evident at six days postinjection and was clearly visible by 11 days postinjection (Figure 1). The brightness of the fluorescence further increased over the following two weeks and then remained stable for the duration of the study. The ssAAV2/5 GFP-injected eyes had weaker GFP expression. GFP expression in these eyes was not apparent ophthalmoscopically until 32 days after injection. GFP expression increased until day 54 and then remained stable for the duration of the study. The strength of GFP expression seen in the histological sections through the injected areas reflected the stronger GFP expression in the scAAV2/5-GFP injected eyes compared to the ssAAV2/5-GFP eyes that was apparent in vivo (Figure 1).

### Cells targeted by ssAAV2/5 and scAAV2/5

**Figure 2:**
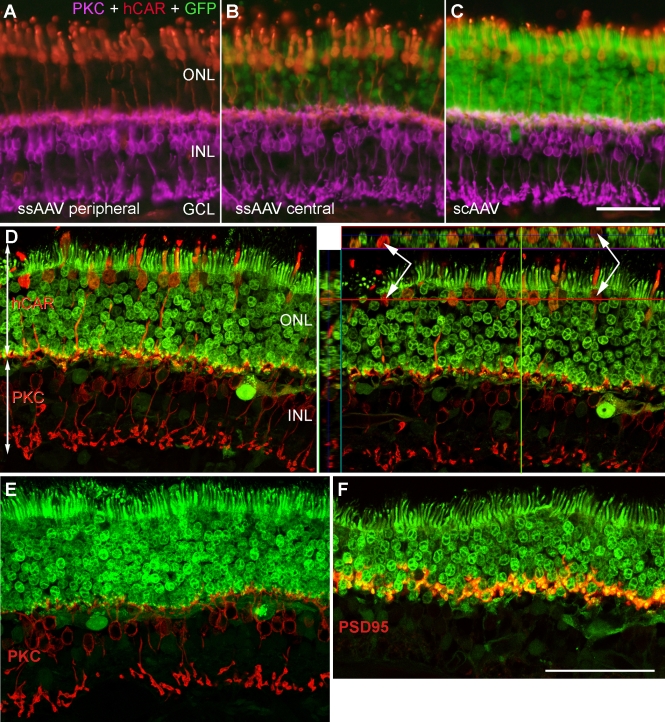
Immunohistochemical investigation of retinal cell type expressing GFP. **A** to **C** are conventional fluorescent microscopy images and **D** to **F** are confocal microscopy images. **A **and **B**: Sections through the ssAAV2/5 injected eye from the peripheral retina away for the injection site (**A**) and through the injection site (**B**). **C**: A section through the injection site in a scAAV2/5 injected eye. **D**: A section through the injected region of a scAAV2/5 eye. The Z stack is shown on the right. Co-localization of hCAR immunoreactivity and GFP expression is present in some, but not all of the cone photoreceptors. Arrows indicate two hCAR positive cones that do not have observable endogenous GFP expression. **E**: The rod bipolar cells (which are PKC immunoreactive) are not expressing GFP. **F**: There is co-localization of GFP expression and PSD95 immunoreactivity in photoreceptor terminals. Abbreviations: ONL, outer nuclear layer ; INL, inner nuclear layer; GCL, ganglion cell layer, PKC, protein kinase C (rod bipolar cell marker); hCAR, human cone arrestin (a cone marker); GFP, green fluorescent protein; PSD95, post synaptic density protein 95 (a marker for rod and cone spherules). Scale bar equals 50 μm.

**Figure 3:**
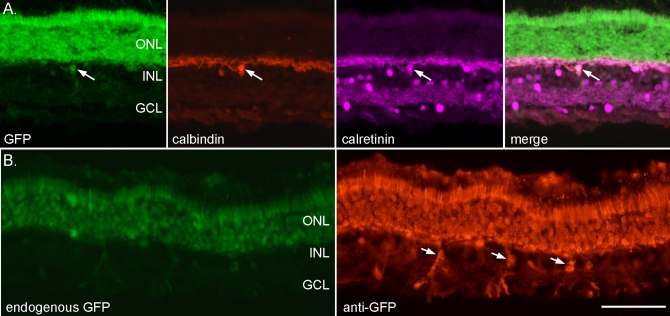
AAV2/5-GFP transduction includes a few horizontal cells and Müller glia. **A**: The panel shows native GFP fluorescence (scAAV2/5-GFP-injected retina), calbindin and calretinin immunohistochemistry and a merge of all three. The arrow indicates a horizontal cell that is expressing GFP. **B**: The image on the left shows endogenous GFP expression; some inner retinal cells are expressing GFP (ssAAV2/5-GFP-injected retina). The image on the right shows immunohistochemistry using an anti-GFP antibody. The arrows indicate Müller cells that are expressing GFP. Scale bar equals 50 µm.

**Figure 4:**
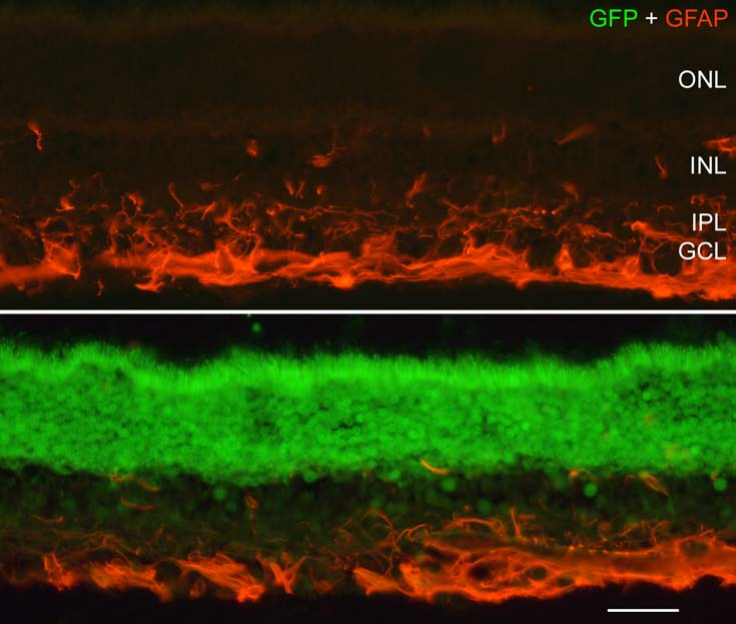
Investigation of GFAP expression in the injected retina. The expression of GFAP was similar between transduced (lower panel) and non-transduced (upper panel) regions of scAAV2/5-injected eyes. The similarity in GFAP immunoreactivity between the injected and non-injected areas indicates that expression of GFP is not inducing glial cell reactivity. Scale bar equals 25 µm.

Retinal pigment epithelium (not shown) and photoreceptors in ssAAV2/5-injected and scAAV2/5-injected eyes expressed GFP (Figure 1B, Figure 2B-F, Figure 3, and Figure 4). Rod cell bodies and inner and outer segments clearly expressed GFP. In both ssAAV2/5- and scAAV2/5-injected eyes some cones in the transduced areas did not express GFP. To further assess cone GFP expression, we used a human cone arrestin (hCAR) antibody that marks the entire cone cell body (Figure 2A-C). This confirmed that within the transduced region, GFP expression was not detectable in all cones (cones not expressing GFP at detectable levels are indicated by arrows in Figure 2D). To assess the proportion of GFP-expressing cones, we counted the number of GFP-positive hCAR immunoreactive cone photoreceptors across sections of the injected area of one of the scAAV2/5 treated retinas. Of 92 consecutive hCAR immunoreactive cones, 55 had clear GFP expression.

Some inner retinal cells were transduced in both scAAV2/5- and ssAAV2/5-injected eyes. Immunostaining with protein kinase C alpha that detects rod ON bipolar cells did not identify any that had been transduced (Figure 2). Using conventional fluorescence microscopy, we found it was not possible to differentiate between expression in photoreceptor end terminals and in bipolar cell dendrites (see Figure 2C). Confocal microscopy provided the resolution to show that GFP expression was absent from rod bipolar cell dendrites (see Figure 2E for an example). Immunostaining with PSD95 confirmed that there was GFP expression in the photoreceptor synaptic terminals (Figure 2F). Further investigation of the inner retinal cells expressing GFP showed that some horizontal cells (Figure 3A) and Müller cells (Figure 3B) were also transduced. Immunostaining with an anti-GFP antibody made it possible to detect inner retinal GFP expression in the ssAAV2/5-injected retinas. The stronger expression of GFP in scAAV2/5-injected retinas meant that they did not require immunostaining for GFP to enhance detection.

Glial fibrillary acidic protein (GFAP) staining did not reveal any appreciable difference in GFAP expression in glia in the transduced regions compared to areas of retina outside of the transduced region in either ssAAV2/5- or scAAV2/5-injected eyes (Figure 4). GFAP immunoreactivity was confined to astrocytes in inner retinal layers. There was no indication of reactive (GFAP expressing) Müller glia.

## Discussion

Subretinal injection of an scAAV2/5 vector in the dog results in stronger reporter gene expression compared to that produced by a single-stranded version of the vector at the same titer and volume of injection. GFP expression could be detected on ophthalmoscopic examination six days after injection in the scAAV2/5-injected eyes compared to 32 days in the ssAAV2/5-injected eyes. Once fluorescence levels peaked, they remained stable for all injected eyes (scAAV2/5 and ssAAV2/5) for the duration of the study. These findings are similar to mouse studies that compared retinal transduction with scAAV2/5 versus ssAAV2/5 vectors [18]. Natkunarajah et al. monitored reporter gene expression by funduscopic examination following injection of an AAV2/8, either with a single copy of the reporter gene (ssAAV2/8) or a sc construct (scAAV2/8), and found stronger reporter gene expression with the scAAV2/8 vector compared to the ssAAV2/8 [19]. Using either ss or sc AAV2-GFP vectors, with fluorescence assessment at the cell-level, Yokoi et al. found earlier and stronger expression in photoreceptors following subretinal injection of the scAAV2 compared to the ssAAV2 [18]. They also demonstrated that GFP expression in retinal pigment epithelium occurred before that in photoreceptors. With the design of our study, it was not possible to assess any difference in the rate of gene expression between different retinal cell types. The difference in rate of GFP expression seen between the scAAV2/5-injected eyes and the ssAAV2/5-injected eyes may be either because of the faster expression expected from an sc construct or as a result of the much stronger expression induced by this vector, or a combination of both factors. The current study only used a single titer of the vectors. It is possible that if the vector titer were increased, the difference in onset and strength of reporter gene expression between the two vector types might be diminished. Additional experiments would be required to investigate this possibility and to optimize vector titer for the two vector types such that all rods and a high proportion of cones were transduced across the injected region. The volume of injection was selected because in previous studies we found that it was sufficient to cause a detachment of about one-quarter of the retina and yet allow the retina to reattach within one or two days postinjection.

Despite uniform strong expression in rod photoreceptors across the injected area, only about 60% of cone photoreceptors expressed GFP. This appears to be a greater proportion of cones than previously recorded in a study using an AAV2 vector expressing GFP under the CMV promoter [22]. In that study, only 5% to 10% of cones were transduced, compared to a high percentage of rods. Although we did not attempt to investigate whether there was a difference in GFP expression between L/M, and S cones, it is of interest that an S-cone promoter that had been successfully used in the rat [23] failed to transduce S-cone photoreceptors in the dog [24]. The proportion of S-cones in the dogs is between 9% and 18% [25]. Thus, it seems unlikely that a lack of expression in S-cones alone accounts for the cones in our study that did not express GFP. Clearly further vector and promoter development is required to optimize the targeting of cones in the dog.

Some inner retinal neurons were found to express GFP in this study, although AAV2/5 has not been reported to target inner retinal cells efficiently [26]. In addition, expression in some Müller cells was seen. Access of the vector to inner retinal cells may have been from the subretinal space or it is possible that there was some reflux of viral particles through the retinotomy site into the vitreous allowing access from the vitreal surface. A previous study delivering AAV2-GFP by subretinal injection in dogs reported ganglion cell expression of GFP. It was thought that the vector reached the ganglion cells due to reflux into the vitreous through the retinotomy site [22]. We found a few examples of transduced horizontal cells, whereas bipolar cells were not transduced.

This study showed that scAAV2/5-GFP delivered by subretinal injection in the dog results in funduscopically detectable GFP protein levels much more rapidly than achieved with an ssAAV2/5 construct. Thus scAAV vectors show promise for the treatment of rapid onset retinal dystrophies where speed of transgene expression may be critical. The stronger level of expression achieved with the scAAV vector may also mean that lower titers of scAAV compared to ssAAV achieve the same level of transgene expression perhaps reducing the risk of adverse effects due to immune responses to the vector itself. Unfortunately, the relatively limited packaging capacity of AAV vectors (typically stated to be 4.7 kb) will limit the size of transgene that can be packaged in a self-complementary manner. However, a recent report suggested that AAV5 capsids could be efficiently packaged with up to 8.9 kb of transgene [27].
